# Recombinant Scorpine Produced Using SUMO Fusion Partner in *Escherichia coli* Has the Activities against Clinically Isolated Bacteria and Inhibits the *Plasmodium falciparum* Parasitemia *In Vitro*

**DOI:** 10.1371/journal.pone.0103456

**Published:** 2014-07-28

**Authors:** Chao Zhang, Xinlong He, Yaping Gu, Huayun Zhou, Jun Cao, Qi Gao

**Affiliations:** 1 School of Basic Medical and Biological Sciences, Medical College of Soochow University, Suzhou, Jiangsu Province, People’s Republic of China; 2 Jiangsu Institute of Parasitic Diseases, Key Laboratory of Parasitic Disease Control and Prevention (Ministry of Health), Jiangsu Provincial Key Laboratory of Parasite Molecular Biology, Wuxi, Jiangsu Province, People’s Republic of China; 3 The Third People's Hospital of Wuxi, Wuxi, Jiangsu Province, People’s Republic of China; Federal University of São Paulo, Brazil

## Abstract

Scorpine, a small cationic peptide from the venom of Pandinus imperator, which has been shown to have anti-bacterial and anti-plasmodial activities, has potential important applications in the pharmaceutical industries. However, the isolation of scorpine from natural sources is inefficient and time-consuming. Here, we first report the expression and purification of recombinant scorpine in *Escherichia coli,* using small ubiquitin-related modifier (SUMO) fusion partner. The fusion protein was expressed in soluble form in *E. coli*, and expression was verified by SDS-PAGE and western blotting analysis. The fusion protein was purified to 90% purity by nickel–nitrilotriacetic acid (Ni^2+^–NTA) resin chromatography. After the SUMO-scorpine fusion protein was cleaved by the SUMO protease, the cleaved sample was reapplied to a Ni^2+^–NTA column. Tricine/SDS-PAGE gel results indicated that Scorpine had been purified successfully to more than 95% purity. The recombinantly expressed Scorpine showed anti-bacterial activity against two standard bacteria including *Staphylococcus aureus* ATCC 29213 and *Acinetobacter baumannii* ATCC 19606, and clinically isolated bacteria including *S. aureus* S, *S. aureus* R, *A. baumannii* S, and *A. baumannii* R. It also produced 100% reduction in *Plasmodium falciparum* parasitemia *in vitro*. Thus, the expression strategy presented in this study allowed convenient high yield and easy purification of recombinant Scorpine for pharmaceutical applications in the future.

## Introduction

The oldest known scorpions lived around 430 million years ago in the Silurian period, on the bottom of shallow tropical seas, hence regarded as the oldest terrestrial arthropods [1]. Scorpions use venoms for immobilization of prey and protection against predators. Scorpion venoms consist of a complex of several toxins that exhibit a wide range of biological properties and actions, as well as chemical compositions, toxicity, pharmacokinetic, and pharmacodynamic characteristics [2].

Scorpions are a rich source of antimicrobial peptides: (1) androctonin isolated from the hemolymph of *Androctonus australis*, shows marked sequence similarity to tachyplesins, polyphemusins and gomesin [3], [4]; (2) hadrurin from the venom of Hadrurus aztecus, which is hemolytic [5]; (3) opistoporin-1, which possesses hemolytic activity, and opistoporin-2, both from the venom of the South African scorpion *Opistophtalmus carinatus* [6]; (4) scorpine, which is the subject of this study, arising from the venom of *Pandinus imperator*, was shown to have anti-bacterial and anti-plasmodial activity *in vitro* [7], and has shown a potent toxic effect on sexual and asexual stages of *Plasmodium berghei* and *Plasmodium falciparum*, respectively, and also a strong inhibition of dengue 2 virus (DENV-2) infection[8]. Native scorpine purified from venom glands has a molecular mass of 8,350 Da. It has a peculiar structure compared to other known AMPs. Its N-terminal amino acid sequence is similar to cecropins, whereas its C-terminal region has several disulfide bridges, similar to the structure of defensins [7]. Scorpine’s wide range of activities provides the possibility that scorpine can be used as an anti-microbial and anti-plasmodial agent in the future.

For pharmaceutical applications, a large quantity of antimicrobial peptides need to be produced economically. Isolation of antibacterial peptides from natural sources is inefficient and time-consuming. The biosynthesis of recombinant antimicrobial peptides *in vivo* is currently the most popular technique of preparing polypeptides. The *Escherichia coli* expression system is still the most commonly used because of its high level of expression, the relative simplicity of the DNA manipulations, and the short time required to produce product. Because of their natural destructive behavior toward microorganisms and relative sensitivity to proteolytic degradation, antimicrobial peptides are often produced by being fused to a fusion partner in heterologous hosts to neutralize their innate toxic activity and increase their expression levels [9].

Small ubiquitin-related modifier (SUMO) is an ubiquitin-related protein that functions by covalent attachment to other proteins. It is known that the SUMO, fused at the N-terminus with other proteins, can fold and protect the protein by its chaperoning properties, making it a useful tag for heterologous expression. These advantages include the manner in which protein expression is enhanced, proteolytic degradation of the target protein is decreased, protein folding and solubility are increased, and purification and detection are simplified [10]–[12].

In this study, we provide the first report of the procedures for obtaining a recombinant Scorpine by using SUMO fusion partner and investigate its anti-bacterial and anti-plasmodial activities.

## Materials and Methods

### Bacterial Strains, Vectors, and Enzymes

*E. coli* DH5a (maintained in our laboratory) was used for subcloning and plasmid amplification. *E*. *coli* BL21 (DE3) (Novagen, USA) was used as the expression host. The linearized pSUMO vector with *Bsa* I and *Xho* I restriction sites and T7 promoter and kanamycin resistance and 6×His sequence was purchased from LifeSensors (LifeSensors, Malvern, PA, USA). SUMO protease containing a histidine-tag was also the product of Lifesensors (Malvern, PA, USA). All the restriction enzymes and T4 DNA ligase were purchased from Takara Biotech Co. Ltd (Dalian, China).

Strains of *Staphylococcus aureus* ATCC 29213 and *Acinetobacter baumannii* ATCC 19606 were purchased from China General Microbiological Culture Collection Center (CGMCC, China) and cultured according to the method provided by CGMCC.

### Clinical Bacteria Sampling and Identification

Strains of *S. aureus* S, *S. aureus* R, *A. baumannii* S, and *A. baumannii* R were isolated in clinc in the Third People's Hospital of Wuxi. The bacteria sampling was obtained in the burn wounds from burn patients, and was crossed to cultivate on blood agar for 24 hours to obtain monoclonal colony, and then monoclonal bacteria were multiplication cultured in liquid medium for 24 h. The identification of proliferative monoclonal bacteria was first carried out with using VITEK^R^ 2 - Compact automatic bacteria identification device (BIOMERIEUX) and furthermore, the isolated bacteria were confirmed through polymerase chain reaction amplification of conserved region of *nuc* gene for *S. aureus* (FW: 5′-GCGATTGATGGTGATACGGTI-3′, RV: 5′-AGCCAAGCCTTGACGAACTAAAGC-3′) [13], and multiplex amplification of 16 S–23 S ribo-somal DNA intergenic spacer region and conserved region of the *recA* gene for *A. baumannii* (FW: 5′-CATTATCACGGTAATTAGTG-3′, RV: 5′-AGAGCACTGTGCACTTAAG-3′ for 16 S–23 S ribosomal DNA intergenic spacer region; FW: 5′-CCTGAATCTTCTGGTAAAAC-3′, RV: 5′-GTTTCTGGGCTGCCAAACATTAC-3′ for *recA* gene) [14]. All PCR products were sequenced to confirm in GenScript Corporation (Nanjing, China).

The antibiotic susceptibility was determined according to the Clinical Laboratory Standards Institute (CLSI) procedure [15]. The minimum inhibitory concentrations (MICs) of selected antibiotics were shown in Table 1. The MICs were determined at concentrations, at which there was no visible growth. The susceptible (S), and resistant (R) strains were defined based on MIC values of <4 µg/ml, and more than 16 µg/ml, respectively [16].

**Table 1:** Minimum inhibitory concentrations of selected antibiotics against *S. aureus* and *A. baumannii*.

Antibiotic	*S. aureus*			*A. baumannii*		
	*S. aureus* ATCC 29213	*S. aureus* S	*S. aureus* R	*A. baumannii* ATCC 19606	*A. baumannii* S	*A. baumannii* R
Ampicillin	<0.25	8	128	256	32	256
Ceftazidime	16	64	256	16	8	64
Gentamycin	0.25	4	256	8	4	256
Amikacin	16	4	256	4	4	256
Erythromycin	0.5	4	256	16	8	256
Tetracycline	0.25	4	64	0.5	0.25	16
Ciprofloxacin	0.25	1	256	4	4	256
Levofloxacin	0.25	4	64	1	0.5	4

Note: Minimum inhibitory concentrations (MIC, µg/ml).

### Ethics Statement

The written informed consents were obtained from the patients in the Third People's Hospital of Wuxi; human red blood cells were obtained from Wuxi Red Cross Blood center (Wuxi, China). This study was approved by the Ethics Committees of the Third People's Hospital of Wuxi and Jiangsu Institute of Parasitic Diseases.

### Construction of Expression Vectors

From the Metarhizium fungus with Scorpine gene of interest, which was a gift from Fang’s laboratory [17], Scorpine gene was amplified by PCR using primers (FW: 5′-TAGGTCTCTAGGTATGGGCTGGATTAACGAGGAGAAG-3′ and RV: 5′-ATTACTCGAGTTAGTAGGAGAGAGGGGTGCC-3′).The PCR fragments were separated using 1.0% gel electrophoresis, purified with a DNA gel extraction kit (Takara, China). The resulting PCR product was digested with *Bsa* I and *Xho* I, and ligated into the pSUMO plasmid at the corresponding restriction sites [18]. The ligation mixture was transformed into *E. coli* DH5a cells for verification by sequencing (Nanjing Genscript Bio. Co. Ltd).

### Expression and Characterization of SUMO Fusion Protein

The pSUMO/Scorpine plasmid that had been constructed, was transformed into competent *E. coli* BL21 (DE3). Three colonies were picked and cultured in 4 ml sterilized Luria–Bertani (LB) medium with vigorous shaking (220 rpm) at 37°C, to an optical density (OD_600 nm_) of 0.6. Isopropyl-b-D-1-thiogalactopyranoside (IPTG) (0.5 mM) was then added to induce the expression of the recombinant protein at 28°C for 8 h.

### SDS-PAGE and Western Blotting Analyses

The SDS-PAGE analysis was performed according to Laemmli [19] using 12% polyacrylamide gels. The total expression protein samples from cell lysates after induction were mixed with equivalent sample buffer (125 mM Tris–HCl, pH 6.8, 20% glycerol, 4% SDS, 0.005% bromophenol blue, and 10% 2-mercaptoethanol). Gels were stained with Coomassie brilliant blue R-250.

The Tricine/SDS-PAGE analysis for the resolution of proteins smaller than 30 kDa was performed according to the reference [20] using 16.5% polyacrylamide gels. Gels were stained with Coomassie brilliant blue R-250.

For western blotting, the same protein sample was separated on a 12% polyacrylamide gel under reducing conditions and then transferred to a polyvinylidene difluoride (PVDF) membrane (Roche Applied Science). The western blotting was performed as described [21]. The rabbit IgG secondary antibody was used against His-tag primary antibody (Novagen). The blots were developed using TMB immunoblotting system.

### Purification of SUMO Fusion Protein

BL21(DE3)-pSUMO/Scorpine strains were cultured in 200 ml sterilized LB medium with vigorous shaking (220 rpm) at 37°C to an optical density (OD_600 nm_) of 0.6. Isopropyl-b-D-1-thiogalactopyranoside (IPTG) (0.5 mM) was then added to induce the expression of the recombinant protein at 28°C for 8 h. Cultures were collected by centrifugation at 12,000×g, at 4°C for 10 min and the cell pellet frozen at −80°C. The pellet from 200 ml culture was then resuspended in 20 ml binding buffer (20 mM Tris, 500 mM NaCl, 20 mM imidazole, and 10 mM phenylmethylsulfonyl fluoride, pH 8.0) and lysed on ice by sonication at 400 W for 100 cycles (4 s working, 8 s free). The supernatant of the cell lysate resulting from centrifugation at 12,000×g at 4°C for 20 min was applied to a nickel–nitrilotriacetic acid (Ni^2+^–NTA, Novagen) affinity chromatography column according to the manufacturer’s instruction. After extensive washing with binding buffer (10 column volumes), the fusion protein was eluted with five column volumes of elution buffer (20 mM Tris, 500 mM NaCl, and 250 mM imidazole, pH 8.0). The peak fractions with high-UV values at 280 nm, which were detected by LPDataView (BIORAD), and containing the fusion protein were pooled and dialyzed overnight at 4°C against phosphate buffered saline (PBS, pH 8.0).

### Purification of Scorpine

The dialyzed fusion protein was reacted with 1 U SUMO protease per 50 µg fusion protein at 30°C for 1 h. Since both SUMO and SUMO protease had 6×His tags, but Scorpine did not, the cleaved SUMO fusion samples could be re-applied to the nickel column to obtain the purified Scorpine by subtracting the 6×His-tagged proteins. Briefly, after the SUMO fusions were cleaved by the SUMO protease, the sample was loaded onto a nickel column with Ni^2+^–NTA resin. Most of Scorpine without 6×His tags was eluted (five column volumes) in the flow-through (unbound) fractions, and the rest was recovered by washing the resin with binding buffer (10 column volumes). The eluted and washed proteins appearing in fractions with high-UV values at 280 nm were pooled as the final purified sample. The purified proteins were checked on SDS-PAGE. Purified Scorpine was filtered through a 0.22 µm filter membrane and stored at −80°C for activity assays.

### Anti-Bacterial Assays

To measure effects of recombinant Scorpine on the growth of bacteria planktonic cell, the bacteria were transferred into 96-well flat-bottomed polystyrene microtiter plates (BD Falcon, SanJose, CA, USA) at approximately 10^5^ CFU/ml in tryptic soy broth (TSB) in the presence of recombinant Scorpine (5 and 10 µM) and cultured at 37°C for 24 h under static conditions, aerobically. After cultivation, the supernatant was 10-fold serially diluted with 0.1% sterile buffered peptone water (BPW), and the dilutions were pour-plated with tryptic soy agar (TSA). The bacteria numbers were determined and calculated by the counts on agar plates.

To measure effects of recombinant Scorpine on the biofilm formation ability of bacteria, the crystal violet (CV) method was used [22]. Briefly, the intact bacteria remainder in each well of the plate after the supernatant transfer was rinsed three times with 0.1% sterile BPW to remove planktonic and loosely attached cells, and the washed wells were air dried at 55°C for 1 h. The dried attached cells were stained with 1% CV solution at 37°C for 30 min, washed twice with sterile distilled water and then air-dried at 55°C for 1 h. The stained biofilm cells were destained with 95% ethanol and measured at 570 nm (OD) (CV_Biofilm_). The negative control (CV_Control_) was used to reduce the background staining from the CV-stained biofilm cells. The biofilm formation ability was expressed by biofilm formation index (BFI = [CV_Biofilm_–CV_Control_]/OD_Planktonic_).

### Anti-Plasmodial Assay

The *P. falciparum* FCC1/HN strain was used for the assay. The FCC1/HN line was isolated from Hainan Island, China [23]. The strain was cultured in human erythrocytes type A+ at 5% hematocrit. The strain was cultured in RPMI (Gibco) medium supplemented with HEPES 25 mM, glutamine 2 mM, glucose 2 g/l, NaHCO_3_ 2 g/l, hypoxanthine 29.25 mg/l, gentamicin 60 mg/l and Albumax 1.6% at pH 7.4. Cultures were kept in 96% nitrogen, 3% CO_2_ and 1% oxygen atmosphere at 37°C, and fresh medium was added every 24 h [24].

Synchronization of parasite cultures with 5% sorbitol (Sigma) was performed as previously described [25]. Briefly, parasites were taken when they were mostly at the ring stage, and then spinned down at 1000 rpm to a pellet; 4 mL of 5% sorbitol (in distilled water) were added onto the pellet and incubated for 45 min at room temperature and at the same time shaken with 2 or 3 times, then centrifuged at 1000 rpm and washed with 3 times in malaria culture medium, and finally, the synchronized parasites at the ring stage were cultured in human erythrocytes type A+ at 5% hematocrit. About 24 h after the synchronized parasites were cultured, the majority trophozoite stage parasites were diluted to 1.5% parasitemia and exposed to recombinant Scorpine (5 and 10 µM) for 24, 48 and 72 h. The medium was discharged, and fresh medium with the appropriate recombinant Scorpine concentration was added every 24 h. The percentage of infected red blood cells was determined by two experienced microscopists using the microscopic examination of thin blood films stained with Giemsa. A total of 5,000 cells from several fields were counted for every thin blood film. Each recombinant Scorpine concentration was tested at least triplicate. Controls with recombinant Scorpine solvent (PBS) and non-treated infected erythrocytes were included [8].

### Statistical Analysis

All experiments were conducted in duplicate for at least three replicates. Results were expressed as means±S.D. Statistical analysis was performed according to Student’s t-test by one-way analysis of variance. Significant difference was taken as *p*<0.05.

## Results

### Plasmid Construction and the Expression of SUMO Fusion Protein

**Figure 1:**
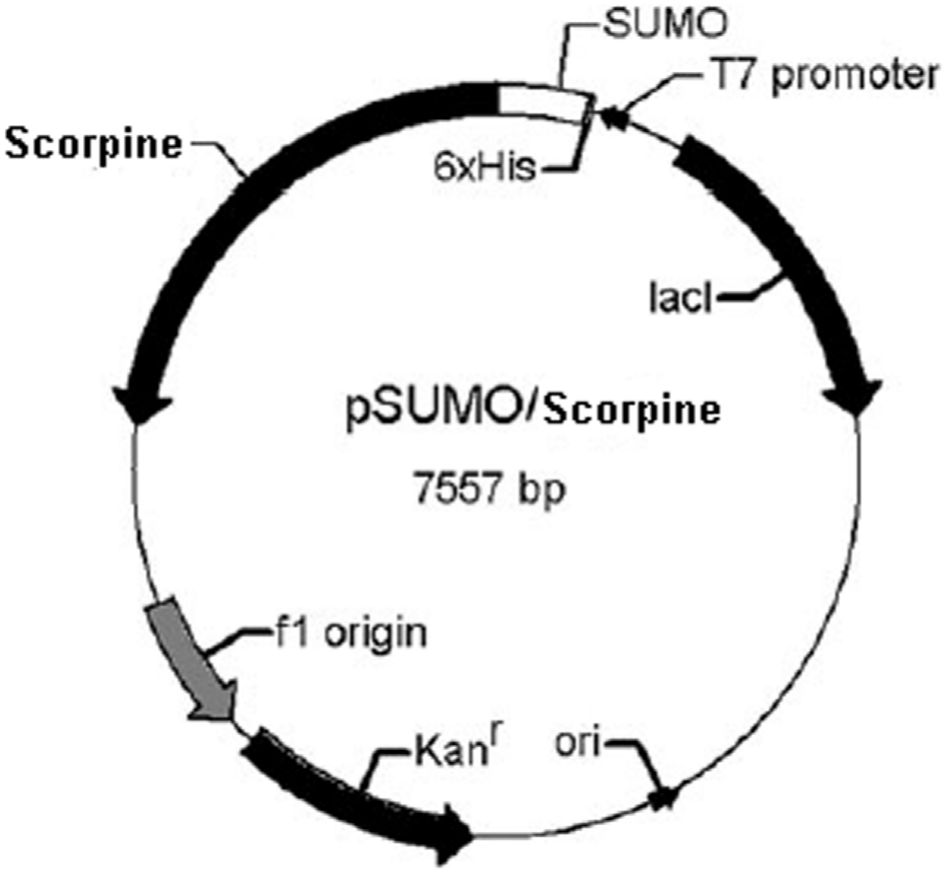
Schematic representation of the expression vector pSUMO/Scorpine. Scorpine was expressed as a fusion protein with the SUMO.

**Figure 2:**
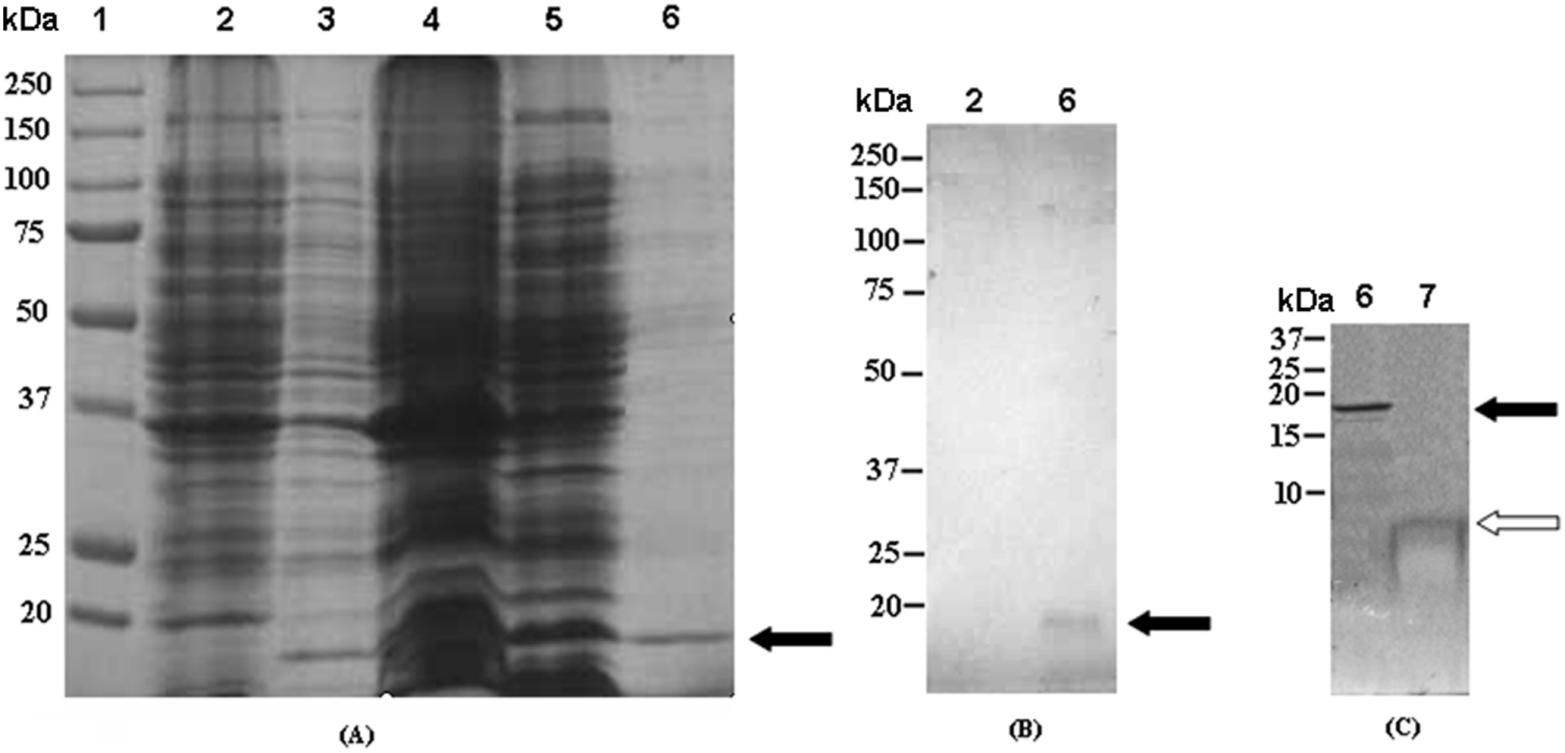
The expression analysis of recombinant Scorpine expressed in *E. coli* BL21. The expression analysis using 12% SDS–PAGE (A), Western blotting (B), and 16.5% Tricine-SDS-PAGE (C). **Lane 1**: Protein molecular weight marker; **lane 2**: Crude cells extracts of uninduced *E. coli* BL21 containing pSUMO/His-Sumo-Scorpine; **lane 3**: Crude cells extracts of induced *E. coli* BL21 containing pSUMO/His-Sumo-Scorpine; **lane 4**: Sonicated precipitate of induced *E. coli* BL21 containing pSUMO/His-Sumo-Scorpine; **lane 5**: Sonicated supernatant of induced *E. coli* BL21 containing pSUMO/His-Sumo-Scorpine; **lane 6**: Purified eluate on a nickel–nitrilotriacetic acid (Ni^2+^–NTA, Novagen) affinity chromatography column; **lane 7**: Scorpine with cleavage of His-Sumo tag using SUMO protease; **Black arrow** showed His-Sumo-Scorpine and **white arrow** showed Scorpine with cleavage of His-Sumo tag.

The construct for Scorpine expression, containing a His-tag for affinity purification, was depicted in Fig. 1. The recombinant plasmid pSUMO/Scorpine sequence was verified by DNA sequencing (Nanjing Genscript Bio. Co. Ltd). The correct construct was transformed into the expression host *E. coli* BL21 (DE3). As shown in Fig. 2A and B, there was an obvious protein band after IPTG induction, which could be detected using the western blotting method with anti-6×His tag antibody. The apparent molecular weight of the SUMO fusion protein was about 18 kDa (the scorpine gene encoded a protein of 75 amino acids, with 8 kDa; and the molecular weight of SUMO is about 10 kDa).

### Purification of SUMO/Scorpine Fusion Protein

As described above, Ni–NTA resin was used for fusion protein purification. Most of the proteins without 6×His tags were removed from the Ni^2+^–NTA resin using washing buffer containing 20 mM imidazole, and the 6×His-tagged SUMO/Scorpine was eluted with more than 90% purity using elution buffer containing 250 mM imidazole (Fig. 2A). The purity was estimated through SDS-PAGE gels stained using Coomassie Blue.

### Purification of Scorpine

The SUMO/Scorpine protein was competently cleaved after incubation with SUMO protease at 30°C for 1 h, according to the method provided by LifeSensors (LifeSensors, Malvern, PA, USA). After the cleaved sample was re-applied to a Ni^2+^–NTA column to remove His_6_-tagged SUMO and SUMO protease, finally purified Scorpine was obtained, and 16.5% Tricine/SDS-PAGE gel results indicated that Scorpine had been purified successfully to more than 95% purity (Fig. 2C).

### Anti-Bacterial Activities of Recombinant Scorpine

**Figure 3:**
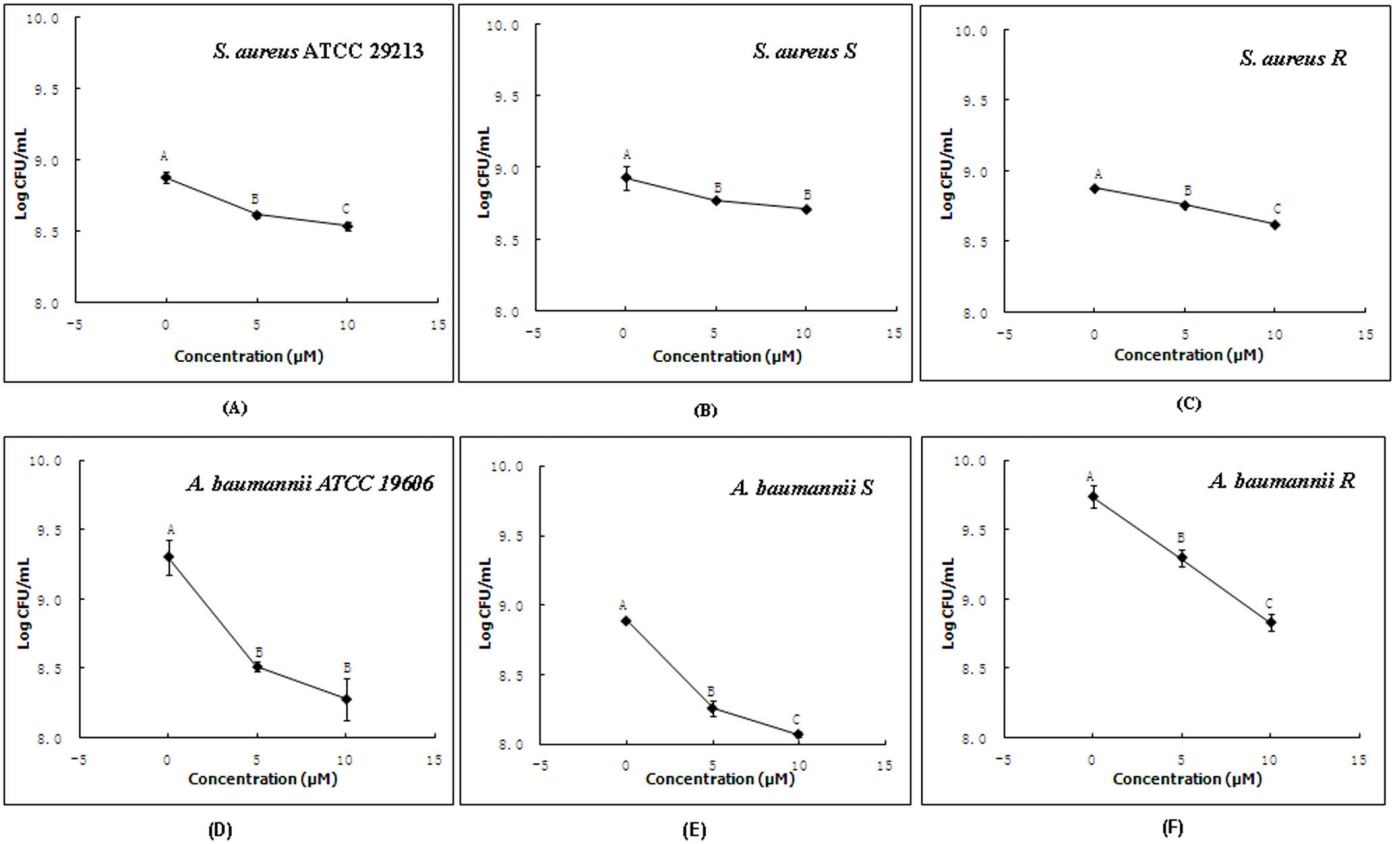
Inhibition of recombinant Scorpine against the growth of planktonic bacteria. (A) *S. aureus* ATCC 29213 (B) *S. aureus* S (C) *S. aureus* R (D) *A. baumannii* ATCC 19606 (E) *A. baumannii* S (F) *A. baumannii* R cultured in the presence of recombinant Scorpine for 24 h. Different letters (A–C) are significantly different within treatments at *p*<0.05.

It was very important to demonstrate that recombinant Scorpine was capable of producing inhibition of bacteria growth similar to that originally shown for native Scorpine as purified from *P. imperator* venom [7]. As shown in Fig. 3, recombinant Scorpine was able to inhibit the growth of two standard bacteria including *S. aureus* ATCC 29213 and *A. baumannii* ATCC 19606 from China General Microbiological Culture Collection Center, and four clinically isolated bacteria including *S. aureus* S, *S. aureus* R, *A. baumannii* S, and *A. baumannii* R.

**Figure 4:**
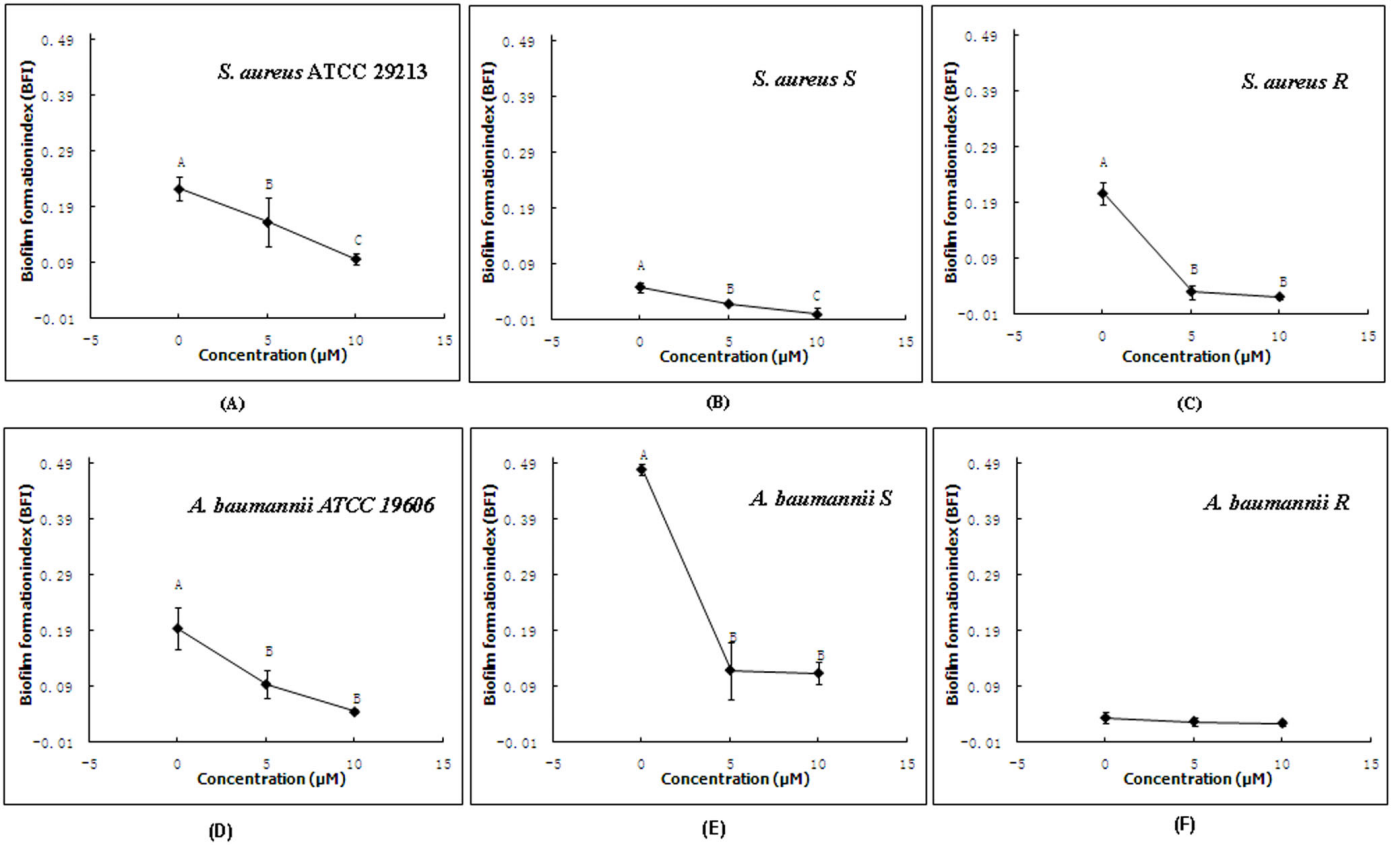
Inhibition of recombinant Scorpine against the biofilm formation indices of bacteria. (A) *S. aureus* ATCC 29213 (B) *S. aureus* S (C) *S. aureus* R (D) *A. baumannii* ATCC 19606 (E) *A. baumannii* S (F) *A. baumannii* R cultured in the presence of recombinant Scorpine for 24 h. Different letters (A–C) are significantly different within treatments at *p*<0.05.

In natural habitats, the biofilm formation is an important survival strategy for bacteria that can be embedded within a self-produced extracellular polymeric matrix of polysaccharides, proteins, lipids and nucleic acids [26], [27]. We investigated effects of recombinant Scorpine on the biofilm formation ability of two standard bacteria including *S. aureus* ATCC 29213 and *A. baumannii* ATCC 19606, and clinically isolated bacteria including *S. aureus* S, *S. aureus* R, *A. baumannii* S, and *A. baumannii* R. As shown in Fig. 4, recombinant Scorpine was able to significantly inhibit the biofilm formation of bacteria, including (A) *S. aureus* ATCC 29213 (B) *S. aureus* S (C) *S. aureus* R (D) *A. baumannii* ATCC 19606 (E) *A. baumannii* S, except that inhibition of recombinant Scorpine against the biofilm formation of (F) *A. baumannii* R did not appear to be significant.

### Anti-Plasmodial Activities of Recombinant Scorpine

**Figure 5:**
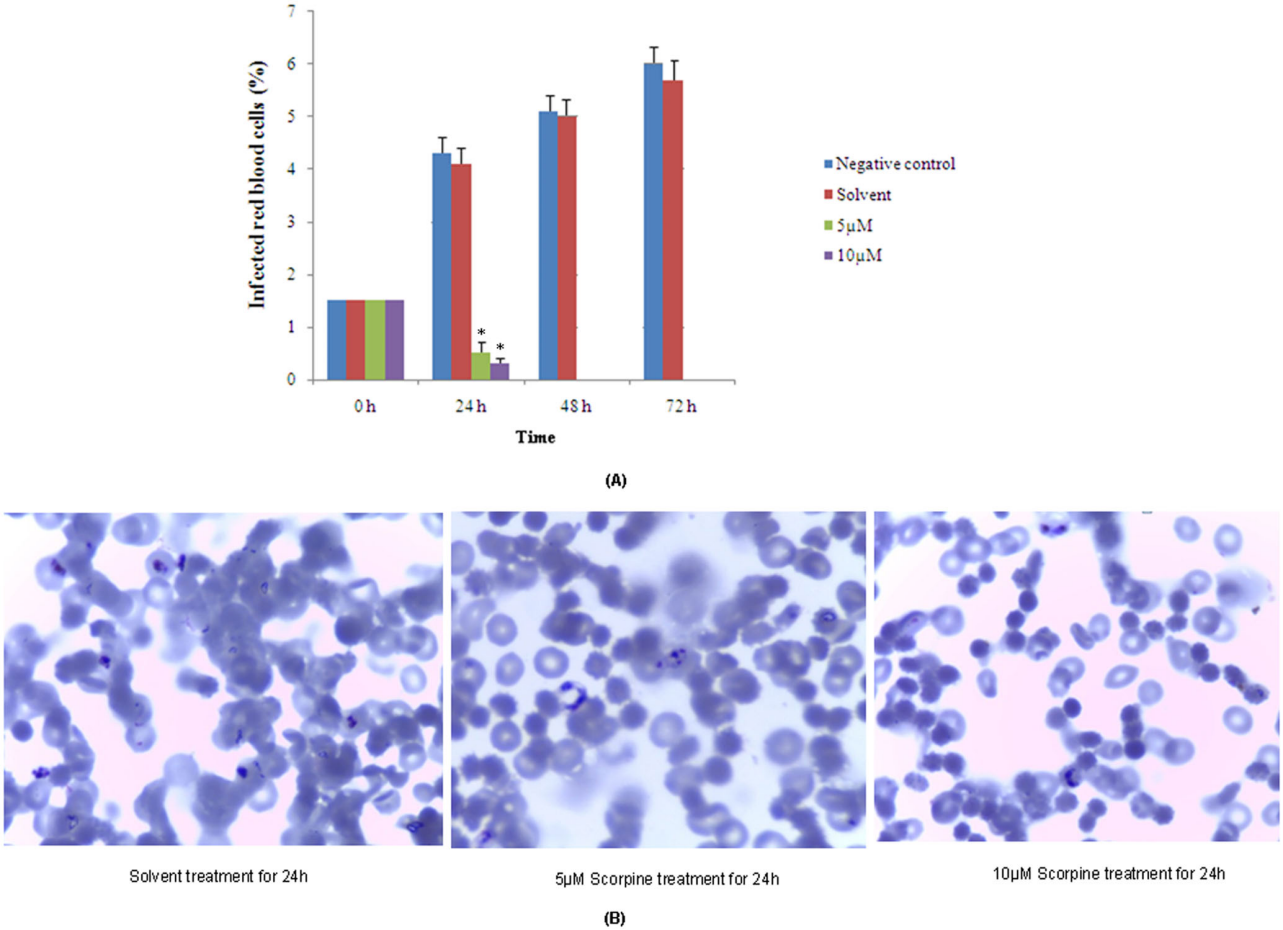
Inhibition of recombinant Scorpine against the infection of *P. falciparum* FCC1/HN *in vitro*. (A) Recombinant Scorpine with cleavage of His-Sumo tag using SUMO protease, was added to *Plasmodium falciparum* FCC1/HN cultures at different concentrations (10 and 5 µM) for 24, 48 and 72 h. The number of infected erythrocytes was counted as described in the “Materials and methods” section. Asterisks indicate significant difference (*p<0.05; comparision with the solvent control). (B) The representative microscopic images of the parasites treated with recombinant Scorpine for 24 h, using the microscopic examination of thin blood films stained with Giemsa (100×, oil immersion).

The trophozoite stage cultures of *Plasmodium falciparum* were exposed to recombinant Scorpine. As shown in Fig. 5 and Fig. S1, the tested concentrations (5 and 10 µM) of recombinant Scorpine reduced *Plasmodium falciparum* parasitemia over the time course of the experiment in relation to controls *in vitro*. And, a critical dependence on exposure time was also observed in recombinant Scorpine treated cultures. At 48 h of exposure, no parasites were observed at recombinant Scorpine 5 µM or above. In addition, no infected erythrocytes were detected after 72 h, at all tested concentrations. During the process of the anti-plasmodial assay in our study, we did not observe that scorpine was hemolytic, at least for recombinant scorpine in *Escherichia coli*. This further suggest that recombinant Scorpine had anti-plasmodial activities.

## Discussion

Scorpion venoms are rich sources of peptides with a variety of pharmacological functions, special those that interact with membrane permeability for Na^+^, K^+^, Ca^2+^ and Cl^–^of excitable and non-excitable cells [28]. Several antimicrobial peptides have been described from scorpions [3]–[5]. Among the peptides isolated from the venom of the African scorpion Pandinus imperator, a molecule, named Scorpine, was identified [7], showing anti-bacterial and anti-plasmodial activities [7], [8], with the possibility of being used as an anti-microbial and anti-plasmodial agent in the future. In this study, we have shown that the expression and purification of recombinant Scorpine in *Escherichia coli,* using the small ubiquitin-related modifier (SUMO) fusion partner.

Small ubiquitin-related modifer (SUMO) is an ubiquitin-related protein that functions by covalent attachment to other proteins. SUMO has 18% sequence identity with ubiquitin [29]. The yeast *Saccharomyces cerevisiae* has only a single SUMO gene (SMT3) that is essential for viability [30]. In contrast to yeast SMT3, three members of SUMO have been described in vertebrates: SUMO-1, SUMO-2, and SUMO-3. Human SUMO-1, a 101 amino acid polypeptide, shares 50% sequence identity with human SUMO-2/SUMO-3 [31], which are close homologues. It is known that SUMO, fused at the N-terminus with other proteins, can fold and protect the protein by its chaperoning properties, making it a useful tag for heterologous expression [12], [32]. All SUMO genes encode precursor proteins with a short C-terminal sequence that extends from the conserved C-terminal Gly–Gly motif. SUMO proteases remove SUMO from proteins, by cleaving the C-termini of SUMO (-GGATY) in yeast to the mature form (-GG) or deconjugating it from lysine side chains [33], [34]. The former activity (protease) is useful for removal of SUMO as an expression tag. In this study, we expressed scorpine as SUMO fusions in *E. coli* to evaluate its anti-bacterial and anti-plasmodial activities. The SUMO fusion protein was successfully expressed in *E. coli*. The SUMO/scorpine fusion protein could be completely cleaved using SUMO protease, as shown in Fig. 2C. The Scorpine, which is about 8 kDa, was recovered with 95% purity using nickel affinity chromatography again.

Similar to that originally shown for native Scorpine as purified from *P. imperator* venom [7], the recombinant Scorpine was able to inhibit the growth of not only standard bacteria from China General Microbiological Culture Collection Center, but also clinically isolated bacteria, suggesting potential important clinical applications (Fig. 3). To further confirm the potential clinical values of Scorpine, we investigated effects of recombinant Scorpine on biofilm formation ability of bacteria, using the CV method [22]. As shown in Fig. 4, the recombinant Scorpine was able to inhibit the biofilm formation of bacteria. The biofilm formation as a bacterial survival strategy leads to increased resistance to heat, acid, preservatives, and antibiotics [35], [36]. Bacterial infections can mainly occur after consumption of contaminated foods. The ingested bacteria are exposed to acidic stress and bile salt under oxygen-limited conditions during transit through the stomach, the small intestine, and the colon. These stress conditions can influence antibiotic resistance patterns, biofilm-forming abilities, and virulence properties [37]. Moreover, antibiotic-resistant bacteria can possibly reside in biofilms and lead to enhanced tolerance to adverse environmental conditions, causing serious infectious diseases [38]–[40]. Our data showed that the recombinant Scorpine was able to inhibit not only the growth of bacteria but aslo the biofilm formation of bacteria, suggest that the potential clinical use of Scorpine in the future.

Malaria, caused by *Plasmodium* infection, is one of the most debilitating parasitic diseases of humans, with an estimated 225 million clinical cases and 781,000 deaths per year [41]. Scorpine was shown to have anti-plasmodial activities [7], [8]. So, we investigated effects of recombinant Scorpine on *P. falciparum*. As shown in Fig. 5, the tested concentrations reduced *Plasmodium falciparum* parasitemia over the time course of the experiment, using *P. falciparum* trophozoite stage cultures that were exposed to recombinant Scorpine. These data suggest that similar to that originally shown for native Scorpine, recombinant Scorpine in *E. coli* was able to inhibit the infection of *Plasmodium spp*.

In summary, the SUMO fusion partner and customized expression and purification protocol described here have further improved the efficiency and lowered the costs of Scorpine production. The SUMO fusion partner could also be widely applied to the industrial production of a variety of enzyme proteins in *E. coli*.

## Supporting Information

Figure S1 The representative microscopic images of the parasites treated with recombinant Scorpine for 0 h, 48 h, and 72 h, using the microscopic examination of thin blood films stained with Giemsa (100×, oil immersion).
(TIF)
