# Supplementary figures and images for: Recombinant Scorpine Produced Using SUMO Fusion Partner in Escherichia coli Has the Activities against Clinically Isolated Bacteria and Inhibits the Plasmodium falciparum Parasitemia In Vitro
Source: PLoS One. 2014 Jul 28;9(7):e103456. doi: 10.1371/journal.pone.0103456 (PMC4113386; doi:10.1371/journal.pone.0103456)

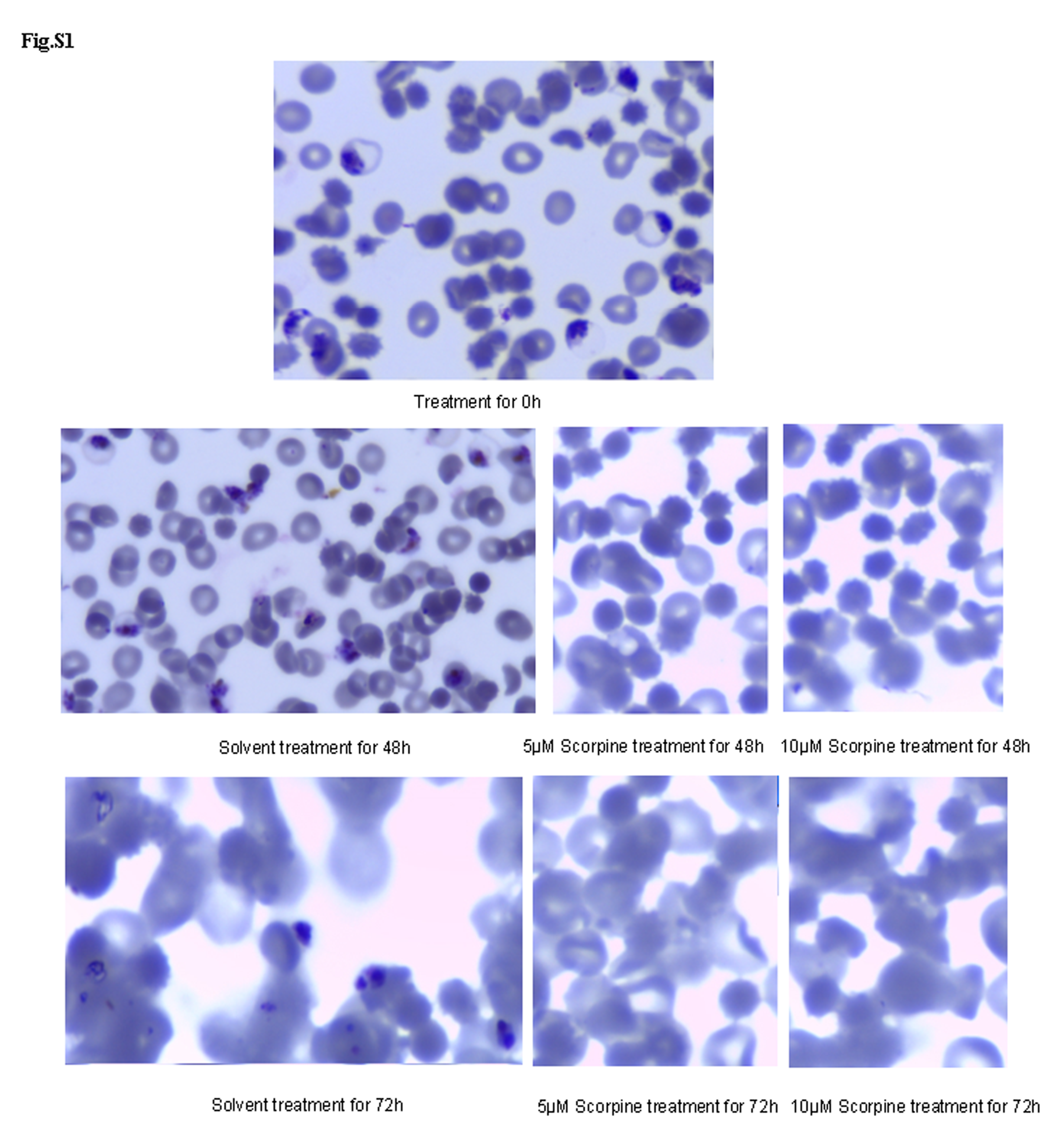

Supplement: Figure S1 — The representative microscopic images of the parasites treated with recombinant Scorpine for 0 h, 48 h, and 72 h, using the microscopic examination of thin blood films stained with Giemsa (100×, oil immersion). (TIF) [file pone.0103456.s001.tif]
